# Systematic review with meta-analysis of diagnostic test accuracy for COVID-19 by mass spectrometry

**DOI:** 10.1016/j.metabol.2021.154922

**Published:** 2022-01

**Authors:** Matt Spick, Holly M. Lewis, Michael J. Wilde, Christopher Hopley, Jim Huggett, Melanie J. Bailey

**Affiliations:** a Faculty of Engineering and Physical Sciences, University of Surrey, Guildford GU2 7XH, UK; b Surrey Ion Beam Centre, University of Surrey, Guildford GU2 7XH, UK; c School of Chemistry, University of Leicester, Leicester LE1 7RH, UK; d National Measurement Laboratory, LGC, Queens Road, Teddington TW11 0LY, UK; e School of Biosciences and Medicine, University of Surrey, Guildford GU2 7XH, UK

**Keywords:** AUROC, Area Under Receiver Operating Characteristic, DI, Direct Injection, FAIMS, high-Field Asymmetric-waveform Ion-Mobility Spectrometry, GC, Gas Chromatography, ESI, Electrospray Ionization, HESI, Heated Electrospray Ionization, IMS, Ion Mobility Spectrometry, LC, Liquid Chromatography, nLC, nano-Liquid Chromatography, MALDI, Matrix Assisted Laser Desorption Ionization, NMR, Nuclear Magnetic Resonance, QTOF, Quadrupole Time of Flight, ROC, Receiver Operating Characteristic, RT-PCR, Polymerase Chain Reaction combined with Reverse Transcription, TFC, Turbulent Flow Chromatography, TOF, Time of Flight, UHPLC, Ultra High pressure Liquid Chromatography, COVID-19, Mass spectrometry, Diagnostics, Meta-analysis, Systematic review

## Abstract

**Background:**

The global COVID-19 pandemic has led to extensive development in many fields, including the diagnosis of COVID-19 infection by mass spectrometry. The aim of this systematic review and meta-analysis was to assess the accuracy of mass spectrometry diagnostic tests developed so far, across a wide range of biological matrices, and additionally to assess risks of bias and applicability in studies published to date.

**Method:**

23 retrospective observational cohort studies were included in the systematic review using the PRISMA-DTA framework, with a total of 2858 COVID-19 positive participants and 2544 controls. Risks of bias and applicability were assessed via a QUADAS-2 questionnaire. A meta-analysis was also performed focusing on sensitivity, specificity, diagnostic accuracy and Youden's Index, in addition to assessing heterogeneity.

**Findings:**

Sensitivity averaged 0.87 in the studies reviewed herein (interquartile range 0.81–0.96) and specificity 0.88 (interquartile range 0.82–0.98), with an area under the receiver operating characteristic summary curve of 0.93. By subgroup, the best diagnostic results were achieved by viral proteomic analyses of nasopharyngeal swabs and metabolomic analyses of plasma and serum. The performance of other sampling matrices (breath, sebum, saliva) was less good, indicating that these protocols are currently insufficiently mature for clinical application.

**Conclusions:**

This systematic review and meta-analysis demonstrates the potential for mass spectrometry and ‘omics in achieving accurate test results for COVID-19 diagnosis, but also highlights the need for further work to optimize and harmonize practice across laboratories before these methods can be translated to clinical applications.

## Highlights

• Mass spectrometry complements RT-PCR in COVID-19 diagnostics.
• Meta-analysis of mass spectrometry diagnostics for COVID-19 shows accuracy of 87%.
• Diagnosis in admissions settings is a promising use-case for mass spectrometry.
• Future work should focus on validating features and protocols across laboratories.

## 1 Introduction

### 1.1 Rationale

The COVID-19 pandemic has resulted in significant morbidity and mortality across the globe [1]. The severity of the pandemic has also triggered developments and accelerated application in many scientific fields, including vaccine technology, drug treatment, and testing. Whilst the global standard in diagnosis has been the polymerase chain reaction combined with reverse transcription (RT-PCR), at times demand has exceeded supply, leading to research across many analytical disciplines for alternative diagnostic solutions [2,3].

The potential of mass spectrometry (MS) for research into diseases and their diagnosis is well-established [4,5], with the flexibility of the technique allowing both proteomic and metabolomic analysis across a wide array of biological matrices. A number of methods have been developed and improved over the last eighteen months [6], but given the exigencies of the pandemic, researchers have often been unable to establish ideal case-controls, blind tests or sufficient participant recruitment to meet best-practice thresholds for either point of care or laboratory-based detection tests [7]. Whilst clinical diagnostic tools such as bilateral chest X-rays and similar methods have been systematically reviewed [2], no such systematic review and diagnostic meta-analysis has to our knowledge been published on tests based on mass spectrometry.

In this review we explored the state of mass-spectrometry-led diagnostic testing for COVID-19 infection across different biological matrices using ‘omics approaches, incorporating a meta-analysis of key parameters. These included accuracy, sensitivity, specificity, and Youden's Index, as well as an assessment of heterogeneity. Any diagnostic test must have a relevant use-case, and in this review we focused on applicability to hospital admissions [8], given that this use-case for MS would complement the capabilities offered by RT-PCR (highly sensitive, but slow turnaround relative to point of care tests) and lateral flow tests (faster but do not take advantage of the facilities and expertise available in a hospital setting). We additionally aimed to assess published studies for issues relating to bias and applicability, in order to review the undoubted progress made so far, as well as to highlight improvements that can be made in future work.

### 1.2 Objectives

The objective of this review was to benchmark a series of MS based diagnostic index tests against each other using RT-PCR as a reference test. We also aimed to identify how well new tests might meet a clinical role of accurate identification of COVID-19 infection, with a focus on admission settings. The review also sought to identify areas in existing research where bias or applicability issues may occur, and how future research may mitigate against these issues.

## 2 Methods

### 2.1 Information sources and search strategy

This study was conducted based on the principles of the Preferred Reporting Items for a Systematic Review and Meta-analysis of Diagnostic Test Accuracy (PRISMA-DTA) statement [9]. Searches were performed in the following databases: Pubmed, Web of Science, Scopus and MedRxiv/BioRxiv. The following terms were required in the search strategy, with alternatives as shown using Boolean operators: “mass spectrometry” AND (“diagnostic” OR “test”) AND (“covid-19” OR “sars-cov-2”). In addition, manual searches were performed for the reference lists of all studies identified by the search strategy described above. The search strategy included articles published on the above-listed databases up to and including 14 September 2021.

### 2.2 Study selection

For all articles identified under the search strategy, titles and abstracts were screened for eligibility. The relevant articles were then read in full, including data extraction for meta-analysis. In this work, the eligibility criteria for inclusion in the systematic review and meta analysis were set as follows: (a) evaluation of a diagnostic method for COVID-19 using mass spectrometry, based on ‘omics approaches, (b) using human biological matrices and (c) including diagnostic analyses, at a minimum reporting sensitivity and specificity by confusion matrix, or receiver operating characteristic (ROC) curves provided that the sensitivity/specificity trade-off was unambiguous. Articles in non-Roman characters were not included.

The above search and eligibility steps were carried out by two researchers, with differences in identified articles reviewed by a third author for inclusion/exclusion.

### 2.3 Data collection process

The following items were collected by two researchers from articles identified above: key metadata for each article (authors, date of publication, country of origin); methods employed (mass spectrometry, separation, biological samples collected) and diagnostic outcomes (true positive – TP; false positive - FP; false negative – FN; and true negative - TN). Diagnostic outcomes were taken directly from research where possible, or were calculated using confusion matrices based on reported sensitivity and specificity outcomes as applied to cohort data, or in one case by use of a reported ROC chart.

### 2.4 Risks of bias and applicability

Two researchers independently evaluated risks relating to both bias and applicability using the Diagnostic Precision Study Quality Assessment Tool (QUADAS-2) [10], with the approach (and conflicts between the researchers) being reviewed by a third author.

### 2.5 Diagnostic accuracy measurements including meta-analysis of diagnostic accuracy

Meta-analysis was performed for the aggregate of mass spectrometry ‘omics based approaches. Given the small sample sizes, not all subgroups offered meaningful results, but subgroups comprising viral proteomics, blood-based metabolomics, and novel ‘omics approaches (saliva, sebum and breath) were reviewed independently from the aggregate. The following ratios were calculated: sensitivity, specificity, diagnostic accuracy, Youden's Index, positive likelihood ratio (PLR) and negative likelihood ratio (NLR).

Sensitivity was defined as the true positive rate, i.e. the probability that a positive test result will be obtained when the disease is present, and calculated as TP/(TP + FN). Specificity was defined as the true negative rate, i.e. the probability that a negative test result will be obtained when the disease is not present, and calculated as TN/(TN + FP). Youden's Index was defined as sensitivity - (1 - specificity), or alternatively, one minus the sum of the error rates. The PLR was defined as the true positive rate/false positive rate. The NLR was defined as false negative rate/true negative rate.

Heterogeneity of diagnostic power across the different biofluids investigated in this work was investigated by measuring Cochran's Q and Higgins I^2^. In this work, a *p*-value below 0.10 or I^2^ value greater than 50% was taken as evidence of substantial heterogeneity of diagnostic power; it should be noted however, that lower values do not necessarily confirm homogeneity, only an absence of evidence for heterogeneity [11,12]. A summary receiver operating characteristic (sROC) curve was also constructed for the studies included herein. ROC curves show the trade-off between sensitivity and specificity, whereby a test can be more sensitive (by over-diagnosing disease) at the cost of being less specific (more false positives), and vice versa. A test that was 100% sensitive and 100% specific would generate an area under the curve (AUROC) of exactly 1, and more generally values closer to 1 indicate better diagnostic performance.

### 2.6 Statistical tools

All statistical analysis was performed in the R Studio environment [13,14], with additional functionality using the epiR, forestplot and mada packages [[15], [16], [17]].

## 3 Results

### 3.1 Study characteristics

**Fig. 1:**
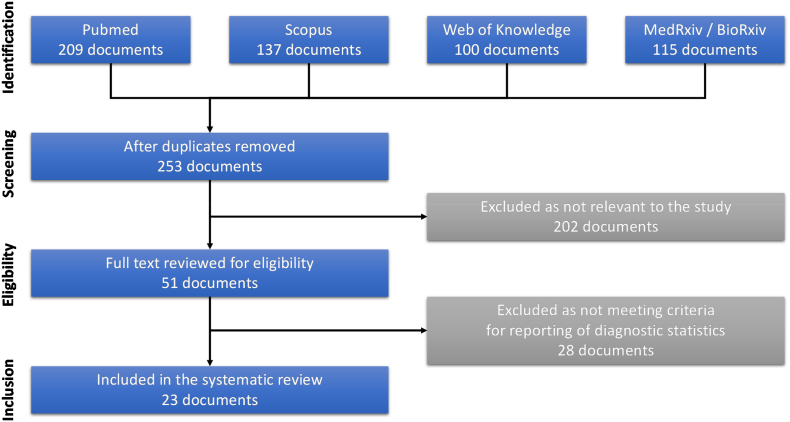
Flowchart of search strategy and results.

In total, 253 articles were identified in the initial search strategy by the terms described, after removing 308 duplicate results. From this initial list, 51 were identified as meeting the eligibility criteria and 202 were excluded. The articles on this shortlist were then read in full. 23 of the 51 identified articles contained the complete set of diagnostic accuracy data to allow for meta-analysis, albeit for one article [18] the data were imputed from provided ROC charts. Fig. 1 provides a flowchart illustrating these steps.

The studies analysed in this review were all conducted in the years 2020–2021, and in total recruited 5402 participants. Reflecting the difficulties of conducting research in a pandemic, all 23 studies were based on retrospective observational cohorts. 11 of the 23 studies separated participants into training and testing groups. Only 1 study included training, testing and blind validation cohorts. The 23 studies were conducted in 12 different countries, mainly in Europe and the Americas, coincident with high prevalence of COVID-19 infection in 2020 and the first half of 2021 [19]. 5 of the studies focused on detection of peptides originating from the virus itself, 3 were untargeted and focused on features irrespective of source, and the remaining 15 analysed host characteristics by a variety of ‘omics techniques. 3 of the studies reviewed were preprints. Further characteristics of the individual studies are summarised in Table 1, grouped by methods whose focus was on host characteristics, methods focused on the virus (by proteomics), and groups that identified features but were agnostic as to the source of those features.

**Table 1:** Characteristics of the included studies, grouped by target and sample type.

Study	Country	Total COVID-19 positive/negative participants	Method	Sample	Main differentiators
Host-targeted approaches					
Maras (2021) [20]	India	120/ 120^a^	LC-MS/MS	Nasopharyngeal Swab	MX1 and WARS proteins
Rocca (2021) [21]	Argentina	123/188^a^	MALDI-TOF-MS	Nasopharyngeal swab	Various *m*/*z* features, non-virus
Yan (2021) [35]	China	146/152	MALDI-TOF MS	Serum	Peptides linked to amyloid fibres, neutrophils and inflammatory/immune response
Garza (2021) [22]	Brazil/USA	74/194	ESI-MS	Nasopharyngeal swab	PE, LysoPE and ceramides
Berna (2021)	USA	22/27	GCxGC-MS	Breath	Octanal, heptanal, nonanal
Ruszkiewicz (2021) [23]	Germany/UK	67/31	GC-IMS	Breath	Ethanal, octanal, acetone, butanone, methanol, isoprene, heptanal, propanol, propanal
Wadah (2021) [24]	UK	52/29	GC-MS	Breath	Benzaldehyde, 1-propanol, 3-6 methylundecane, camphene, beta-cubene, iodobenzene
Grassin-Delyle (2020) [25]	France	28/12	Heated transfer line to QTOF MS	Breath	Methypent-2-anal, 2,4-octadiene, 1 chloroheptane, nonanal
Fraser (2020) [18]	Canada	10/20^c^	DI-MS/LC-MS/MS plus NMR	Plasma	Arginine/kurenine ratio and creatinine
Delafiori (2020) [26]	Brazil	442/373^a^, ^b^	HESI-MS	Plasma	Cholesterol/LysoPCs
Kimhofer (2020) [27]	Australia	17/25	UHPLC-MS plus NMR	Plasma	Kynurenine/tryptophan ratio, glutamine/glutamate ratio
Gray (2021) [28]	Australia/Spain	332/159	UHPLC-MS	Plasma	Lipid panel (PE, PL, LPC, HCER, CER, DCER)
Spick (2021) [29]	UK	30/37	UHPLC-MS	Skin swab	Odd-chain triglycerides
Delafiori* (2021) [30]	Brazil	64/37	HESI-MS	Skin swab	Oleamide, N-acylethanolamines, N-acylaminoacids, glycerolipids
Frampas* (2021) [31]	UK	47/28	UHPLC-MS	Saliva	Phenylalanine, unidentified *m/z*

Untargeted approaches					
Tran (2021) [32]	USA	107/92^d^	MALDI-TOF-MS	Nasopharyngeal swab	Various *m/z*, not identified
Nachtigall (2020) [33]	Chile	211/151	MALDI-MS	Nasopharyngeal swab	Various *m/z*, not identified
Deulofeu (2021) [34]	Spain	60/176^a^	MALDI-TOF-MS	Nasopharyngeal swab	Various *m/z*, not identified

Virus-targeted approaches					
Cardozo (2020) [36]	Brazil	540/445^a^	TFC-MS	Nasopharyngeal swab	Virus proteins
Chivte (2021) [37]	USA	30/30	MALDI-TOF-MS	Saliva	Virus spike protein S2
Hober* (2021) [38]	Sweden	48/40	UHPLC-MS	Nasopharyngeal swab	Virus nucleocapsid proteins
Renuse (2021) [39]	India/USA	204/159^a^	FAIMS-PRM	Nasopharyngeal swab	Virus nucleocapsid proteins
Singh (2020) [40]	India	83/20^a^	nLC-MS	Nasopharyngeal swab	Virus spike glycoprotein, replicase polyprotein

*Pre-print at the time of writing.

a Total participants across testing, training and (where included) validation subsets.

b Negative participants include 23 COVID-19 suspicious.

c Negative participants comprise 10 COVID-19 negative participants with ARDS and 10 healthy controls.

d Excluding a total of 27 samples invalidated due to polymer contamination.

### 3.2 Risk of bias and applicability of the tests reviewed

**Fig. 2:**
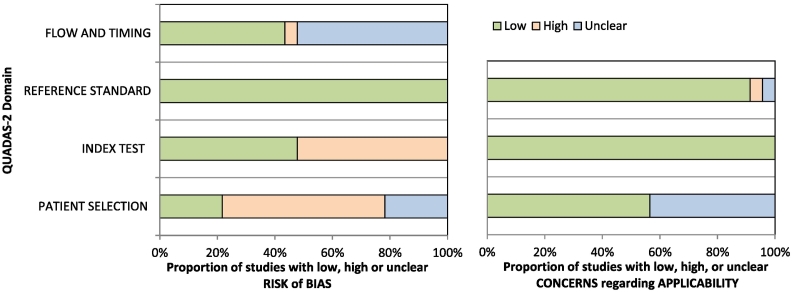
Proportion of studies with low, high or unclear risks of bias or concerns over applicability.

Table 2 summarises identified risks of bias or concerns around applicability for the studies reviewed in this work, established using the QUADAS-2 framework, with the proportion of studies by each risk category shown in Fig. 2. The questionnaire designed for this review is included within the Supplementary Material. A number of inherent issues present themselves due to the desire to conduct research rapidly in a pandemic situation, and it should be noted that all the studies reviewed here recognised these difficulties.

**Table 2:** Risks relating to bias and to applicability.

	Bias				Applicability		
	Patient selection	Index test	Reference standard	Flow and timing	Patient selection	Index test	Reference standard
Berna (2021)	High	High	Low	Unclear	Low	Low	Low
Cardozo (2020)	High	Low	Low	Low	Unclear	Low	Low
Chivte (2021)	High	High	Low	Unclear	Unclear	Low	Low
Delafiore (2020)	Low	Low	Low	Unclear	Low	Low	Low
Delafiore (2021)	High	High	Low	Unclear	Low	Low	Low
Deulofeu (2021)	Unclear	Low	Low	Low	Unclear	Low	Low
Fraser (2020)	Low	High	Low	Unclear	Low	Low	Low
Frampas (2021)	Low	High	Low	Low	Low	Low	Low
Garza (2021)	High	High	Low	Low	Low	Low	Low
Gray (2021)	High	Low	Low	Unclear	Low	Low	Low
Grassin-Delyle (2020)	High	High	Low	Unclear	Low	Low	Low
Hober (2021)	High	High	Low	Low	Unclear	Low	Low
Kimhofer (2020)	High	High	Low	Unclear	Unclear	Low	Unclear
Maras (2021)	High	Low	Low	Unclear	Low	Low	Low
Nachtigall (2020)	Unclear	Low	Low	Low	Unclear	Low	Low
Renuse (2021)	Unclear	Low	Low	Low	Unclear	Low	Low
Rocca (2020)	Unclear	Low	Low	Low	Unclear	Low	Low
Ruszkiewicz (2021)	High	High	Low	High	Low	Low	High
Singh (2020)	High	Low	Low	Unclear	Unclear	Low	Low
Spick (2021)	High	High	Low	Unclear	Low	Low	Low
Tran (2021)	Low	Low	Low	Low	Unclear	Low	Low
Wadah (2021)	Unclear	High	Low	Unclear	Low	Low	Low
Yan (2020)	Low	Low	Low	Unclear	Low	Low	Low

In the analysis that follows, Unclear does not denote ‘medium’ risk of concern; rather it denotes that insufficient information was provided, and there is no basis to consider the study to be at ‘low’ risk of bias or inapplicability.

In terms of risks of **bias around patient selection,** 30% of the studies provided no cohort analysis, making it impossible to ascertain whether the work was free from bias in this regard. Furthermore, only 9% studies specified whether participants were recruited consecutively or at random. Only 23% of studies explicitly stated that asymptomatic patients were included, 39% stated that they were excluded, and 39% provided no information, potentially biasing results. Overall, 57% of the studies were assessed as high risk of bias in the patient selection domain, and 22% unclear, with only 22% studies assessed as at low risk of bias.

In terms of **applicability of patient selection**, 61% of the studies reviewed in this report stated explicitly that participant recruitment took place in a hospital setting with 17% obtaining samples from a biobank, and 22% not stating recruitment location. Hospital-based participant recruitment is relevant if MS based testing is considered to have a use-case as a clinical admissions tool. Although hospital recruitment can introduce selection bias by providing the index test with participants with high viral load and low incidences of asymptomatic disease, from an applicability perspective a hospital setting mirrors application in a clinical setting for use on admission for triage or allocation of patients to COVID-19 specific wards. 57% were considered low risk for applicability of patient selection (hospital-based recruitment) and the rest were assessed as of unclear risk.

Looking at **risks of bias in the index test**, 52% of studies operated with separate training and test/validation tests. 48%, however, did not, introducing bias due to test parameters being defined to maximise sensitivity, specificity and AUROC. Due to overfitting, this can lead to sensitivity and specificity being overstated versus the results that could be obtained in a blind validation population. In addition, one of the studies split the sample population into too small a subgroup for appropriate analysis. Consequently, 52% of studies were assessed as high risk in the index test domain and 48% as low risk. Because all tests reviewed here had diagnosis of COVID-19 as their primary objective, no issues relating to **applicability of the index test** were identified.

Regarding the **risks of bias to the reference test**, the studies reviewed in this work used RT-PCR as the gold standard. Mass spectrometry approaches may not be as clinically sensitive as RT-PCR, but offer an alternative approach potentially capable of a faster route to identify more at risk individuals (who have higher viral burden) and support clinical decisions. As many diagnostic laboratories have the technology to perform such analyses. Their use would also capitalise on this alternative established capacity. All 23 studies were considered to be of uniformly low risk of bias with regard to appropriateness of the reference test. Similarly, in terms of applicability, the reference test has as its primary purpose the diagnosis of COVID-19 positive status in participants, and so the **risk of inapplicability of the reference test** was deemed to be low in the reference test domain for all but two studies, which did not uniformly use RT-PCR as the reference standard for all participants and substituted alternatives in a small number of cases.

Finally, in terms of **flow and timing**, whilst all participants in all but one of the studies reviewed received the same reference tests, only 9% of studies specified the time difference between index test and reference test, but with one of these the interval was too large to be considered a low risk. 43% of studies were classified as low risk due to nasopharyngeal swabs being used for both the index MS-based test and the RT-PCR test, making the timing of both tests simultaneous. The remaining 52% of studies were classified as unclear risk in the flow and timing domain.

### 3.3 Diagnostic results of the studies

The key extracted diagnostic indicators are summarised in Table 3 below, including estimated 95% confidence intervals for sensitivity and specificity [41]. Several studies reviewed here presented multiple datasets for different purposes, e.g. testing versus training, or contrasting COVID-19 positive with negatives including or excluding asymptomatic participants. The data in Table 3 represent the results from blind or validation tests where available, or from a training set if this was the only dataset reported. In the event of multiple experiments, the data set at lowest risk of bias or greatest applicability was selected for analysis, concordant with the risk assessment in the previous section.

**Table 3:** Summary of diagnostic indicators, listed by target then by methodology.

Study	Method	*n* employed in diagnostic model	Sensitivity (95% CI)	Specificity (95% CI)	Likelihood ratio (pos/neg)
Host-targeted approaches					
Maras (2021) [20]	Proteomics - Host	200^b^	0.87 (0.79, 0.93)	0.88 (0.80, 0.94)	7.2/0.2
Rocca (2021) [21]	Proteomics – Host	144^b^	0.62 (0.49, 0.73)	0.72 (0.62, 0.80)	2.2/0.5
Yan (2021) [35]	Proteomics – Host	100^b^	0.98 (0.89, 1.00)	1.00 (0.93, 1.00)	NA/0.02
Garza (2021) [22]	Lipidomics - Host	171^b^	0.82 (0.67, 0.92)	0.77 (0.69, 0.84)	3.6/0.2
Berna (2021)	Breathomics – Host	24^b^	0.91 (0.62, 1.00)	0.75 (0.43, 0.95)	3.7/0.1
Ruszkiewicz (2021) [23]	Breathomics – Host	98	0.84 (0.66, 0.95)	0.79 (0.67, 0.88)	4.0/0.2
Wadah (2021) [24]	Breathomics – Host	81	0.68 (0.53, 0.80)	0.85 (0.68, 0.96)	4.9/0.4
Grassin-Delyle (2020) [25]	Breathomics – Host	28	0.90 (0.65, 0.99)	0.94 (0.62, 1.00)	10.7/0.1
Fraser (2020) [18]	Metabolomics – Host	20^c^	0.80 c (0.44, 0.97)	1.00 c (0.69, 1.00)	NA/0.2
Delafiori (2021) [26]	Metabolomics – Host	281^a^	0.83 (0.78, 0.88)	0.96 (0.86, 1.00)	20.8/5.3
Kimhofer (2020) [27]	Multi-omics – Host	18^b^	1.00 (0.72, 1.00)	1.00 (0.59, 1.00)	NA/0.0
Gray (2021) [28]	Lipidomics - Host	206^b^	0.95 (0.90, 0.98)	0.92 (0.81, 0.98)	12.1/0.1
Spick (2021) [29]	Skin Lipidomics – Host	67	0.79 (0.70, 0.87)	0.83 (0.74, 0.90)	4.7/0.3
Delafiori* (2021) [30]	Skin Lipidomics – Host	101^b^	0.74 (0.61, 0.84)	0.82 (0.65, 0.92)	3.9/0.3
Frampas* (2021) [31]	Saliva Metabolomics	75	0.77 (0.62, 0.88)	0.75 (0.55, 0.89)	3.1/0.3

Untargeted approaches					
Deulofeu (2021) [34]	Untargeted – Host/Virus	84	1.00 (0.92, 1.00)	0.92 (0.79, 0.98)	13.0/0.0
Nachtigall (2020) [33]	Untargeted – Host/Virus	362	0.95 (0.91, 0.97)	0.93 (0.87, 0.96)	13.0/0.1
Tran (2021) [32]	Untargeted – Host/Virus	117^b^	1.00 (0.95, 1.00)	0.96 (0.86, 1.00)	25.0/0.0

Virus-targeted approaches					
Cardozo (2020) [36]	Proteomics – Virus	108^b^	0.84 (0.71, 0.92)	0.93 (0.83, 0.99)	13.3/0.2
Chivte (2021) [37]	Proteomics/Virus	60	1.00 (0.88, 1.00)	0.93 (0.78, 0.99)	15.0/0.0
Hober* (2021) [38]	Proteomics/Virus	88	0.83 (0.70, 0.93)	1.00 (0.91, 1.00)	NA/0.2
Renuse (2021) [39]	Proteomics/Virus	176^b^	0.98 (0.92, 1.00)	1.00 (0.96, 1.00)	NA/0.0
Singh (2020) [40]	Proteomics - Virus	83^b^	0.90 (0.80, 0.96)	1.00 (0.83, 1.00)	NA/0.1

*Pre-print at the time of writing.

a Blind test set participants only.

b Validation test set participants only.

c On ROC curve. NB 80% sensitive and 100% specific also exists on the curve but has equivalent accuracy.

Across the studies reviewed, sensitivity ranged from 0.62 to 1.00 (aggregate sensitivity of 0.87), and specificity ranged from 0.72 to 1.00 (aggregate specificity of 0.88). Specificity was greater than sensitivity on average, albeit the difference was not statistically significant based on a two-tailed *t*-test (*p*-value of 0.34).

In terms of biofluids analysed, sebum was analysed in 2 papers, and delivered the lowest aggregated sensitivity (0.76) and specificity (0.82), calculated by summing confusion matrices. Saliva was investigated in 2 studies, with sensitivity and specificity of 0.74/0.75 for metabolomic analysis of saliva, and 1.00/0.93 for proteomic analysis. Breath was analysed in 4 studies, with comparable sensitivity (0.78) and specificity (0.81) to sebum. Nine (9) studies sampled nasopharyngeal swabs, with high sensitivity (0.89) and specificity (0.88). The remaining 5 studies sampled blood (either plasma or serum), with aggregated sensitivity of 0.89 and specificity of 0.96. Proteomic approaches that targeted the virus reported higher sensitivity and specificity than approaches that targeted the impact on the host, albeit within the latter category there was considerable variation.

Table 1 lists the major features differentiating the populations by study. In studies focusing on proteomics, a number identified features by *m/z* only, but 2 studies targeted peptides originating from spike proteins, and 2 identified peptides originating from nucleocapsid proteins. For the 4 studies analysing breath, a wide variety of alcohols, aldehydes and ketones were found to differentiate the populations, but there was limited overlap, with heptanal and octanal featuring in 2 of the 4 studies. In terms of sebomics, the studies described in this review found no differentiating features in common. Within plasma and serum, 2 papers identified ratios of amino acids (kynurenine in particular) as key differentiating features, and 2 papers focused on lipid dysregulation.

**Fig. 3:**
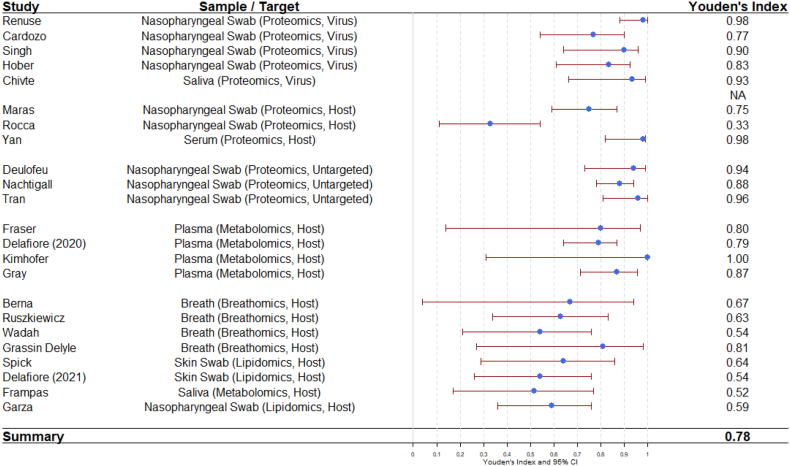
Youden's Index for mass spectrometry techniques applied to the diagnosis of COVID-19: Points represent Youden's Index with horizontal lines indicating 95% confidence interval.

As a single measure of performance, estimates of Youden's Index including confidence intervals are shown in Fig. 3, with Youden's Index calculated as sensitivity minus (one minus specificity), or alternatively one minus the sum of error rates.

### 3.4 Heterogeneity assessment of the studies

The studies show variation in their diagnostic performance measured by either sensitivity, specificity or Youden's Index (Table 2 and Fig. 3) and - partly due to small participant populations - confidence intervals are wide. Cochrane's Q was calculated as 26.2 with a *p*-value of 0.24, and Higgins' I^2^ was calculated as 16%. The latter value should be treated with caution given the small samples sizes assessed in this meta-analysis as Higgins I^2^ tends to be underpowered in the meta-analysis of studies with small *n* and therefore lower precision [42]. A low I^2^ does not represent evidence of homogeneity per se, but may indicate that the variability in results could be due to wide confidence intervals rather than unexplained heterogeneity, as is this case in this work (Fig. S1, Supplementary Material).

Heterogeneity was also investigated by broad method employed, specifically proteomics versus metabolomics, and also by subgroup. Heterogeneity was notably low for proteomics including viral proteins, with Cochrane's Q calculated as 7.6, and Higgins' I^2^ was 0%. For blood-based analyses, Cochrane's Q was calculated as 4.1, and Higgins' I^2^ was calculated as 3%. For saliva, sebum and breath (the more novel ‘omics analyses), Cochrane's Q was calculated as 3.2 and Higgins’ I^2^ was calculated as 0%.

**Fig. 4:**
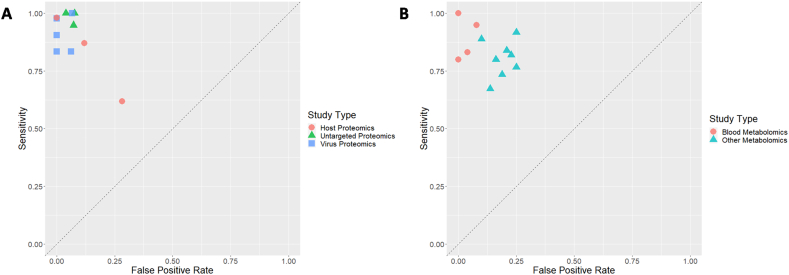
Sensitivity versus false positive rate: (A) Proteomics studies (b) Metabolomics studies (including other ‘omics based on saliva, sebum and breath).

Visual inspection also illustrates the differences between, but similarity within, these methods (Fig. 4A and B). This can also be illustrated by calculating summary area under the sROC curves for these groups. For the aggregate of studies described here, the area under the sROC curve was 0.93, but for proteomic studies targeting the virus plus untargeted studies, the area under the sROC curve was 0.94. For blood-based metabolomic studies, the area under the sROC curve was 0.97, and for ‘omics analyses of other sampling matrices the area under the sROC curve of 0.84 was markedly below other methods.

## 4 Discussion

For the studies considered in this work, there were variations in diagnostic metrics (Fig. 3), but all the studies reported here show potential diagnostic efficacy measured by sensitivity and specificity, with weighted means of 0.87 and 0.88 respectively and an area under the SROC curve of 0.93 (Fig. S2, Supplementary Material). This evidence confirms that there are distinct and identifiable disruptions to pathways across the metabolome and proteome [43,44], and that these host-derived disruptions can be detected, just as the virus itself can be detected.

It should be noted, however, that the features identified in the studies reviewed here showed limited commonality, unsurprisingly given the varied sampling matrices and methods employed. Within subgroups of study, viral proteomics showed the greatest agreement in differentiating features, via both spike and nucleocapsid proteins [45]. Several blood-based metabolomics analyses also showed consistency with reported dysregulation of amino acid and lipid metabolism [46]. The greater heterogeneity of features in metabolomics analyses applied to other sampling matrices (saliva, breath, sebum) may partly reflect instrumental setup, but could also relate to confounders, and illustrates the need for much more inter-laboratory validation and comparison before these diagnostic techniques are likely to be suitable for translation to clinical practice.

RT-PCR as a reference standard achieves very high analytical sensitivity and specificity and is generally seen as the clinical gold standard for release of patients from isolation [47], but there has also to be a role for less sensitive, faster approaches to support a triage environment, e.g. for ward allocation on hospital admission, where a negative RT-PCR result will often require additional testing for confirmation [48]. Antigen detection assays can offer an alternative to RT-PCR with faster response time, depending on type; one meta-analysis found sub-category sensitivity ranging from 0.66 (for lateral flow immunoassays) to 0.98 (for chemiluminescent immunoassays) [49]. Bilateral chest X-rays have also been reported to be a useful supplementary tool in COVID-19 diagnosis. In a recent meta-analysis chest X-rays were found to have sensitivity of 0.91 and specificity of 0.78, again with RT-PCR as the reference [50], albeit the American College of Radiographers has noted that chest imaging in COVID-19 is not specific, and overlaps with other infections [51].

Compared with these benchmarks, MS-based approaches show promise based on achieved sensitivity and specificity and - given that mass spectrometry facilities are often available in hospital settings - may find a use-case by offering faster turnaround than RT-PCR and so supplementing clinical diagnosis. In addition, MS-based approaches offer alternatives in the initial stages of a pandemic, when supplies for PCR or other tests may be in short supply. Because of the ability of MS based techniques to identify dysregulation involving many pathways, such tests could provide information on the wider host metabolome and proteome. This potentially allows for prognosis as well as diagnosis, and promising results have already been obtained for mass spectrometry-based prognostic analyses of serum, plasma and saliva [31,52,53].

In this work, the best results were found to be delivered by metabolomic study of homeostatically regulated biofluids (serum and plasma) and by proteomic study of nasopharyngeal swabs, with areas under their respective sROC curves of xxx and xxx respectively. These results were mainly obtained by UHPLC-MS (for blood metabolomics) and MALDI-TOF-MS (for proteomics). These sampling methods are, of course, more invasive than skin swabs or exhaled breath, but based on the studies reviewed here, the invasive methods deliver the greatest diagnostic accuracy and are most concordant with the WHO's Target Product Profile, which targets 90% sensitivity and 99% specificity as desirable for point of care tests [54]. At this stage, therefore, we view blood based metabolomics and viral proteomics using nasopharyngeal swabs as the most promising in terms of likely clinical application in the near term.

Whilst other ‘omics approaches may offer alternative solutions that are less invasive, results from alternative matrices show lower sensitivity and specificity and a lack of commonality of features identified. This heterogeneity suggests that considerable further work is needed in validation and optimisation. A further caveat is that host-based approaches have not been tested in an environment with widespread respiratory viruses, which could challenge specificity [55,56]. Of course, host ‘omics also offer the potential for additional insight into patient health above and beyond diagnosis of the virus, albeit prognostic ‘omics studies face further challenges due to the varied phenotypes presented during COVID-19 infection [57].

In addition, other limitations in the works reviewed are evident relating to bias and applicability, and the studies reviewed here were hampered by the natural difficulties in conducting research in a pandemic setting. Biases relating to patient selection and to the creation of separate testing and training sets for machine learning were common in the work described herein, reflecting the exigencies of the pandemic and acknowledged by all the articles reviewed. An overarching issue is the small sample sizes. As well as limiting the ability to create separate testing and training sets, the numbers recruited typically fall short of best practise for developing diagnostic tests, such as the Target Product Profile set by the UK Department for Health and Social Care which mandates testing in the validation set of 150 COVID-19 positive and 250 COVID-19 negative participants [7]. Whilst statistical analysis shows low heterogeneity of diagnostic performance for proteomics and blood-based sampling, the variation (and lack of overlap) in differentiating features suggests that much more inter-laboratory validation and optimisation will be required before these results can be translated into a clinical setting. The pilot studies described herein have shown the potential for accurate diagnosis of COVID-19, but we believe that future work should focus on larger recruitment cohorts, the inclusion of more blind tests for validation, validation across different locations, and optimisation of techniques.

## 5 Conclusions

The detection and diagnosis of COVID-19 by mass spectrometry has made substantial progress over the course of the SARS-CoV-2 pandemic. Achieved sensitivity and specificity of the diagnostic tests discussed in this review are encouraging, but with clear limits in the biases and applicability of the research undertaken so far. Whilst results based on proteomics and blood metabolomics delivered the most compelling performance, and these methods are most promising in terms of clinical application in the near term, more validation studies are still needed to reduce risks of bias and applicability. In the case of less invasive matrices, whilst the potential advantages are attractive, as yet there is little agreement between studies on suitably robust and reproducible targets.

Whilst mass spectrometry techniques may show promise, and advances in this field could be applicable to disease diagnosis beyond COVID-19, future research should focus on reducing bias by recruiting larger numbers of participants without inappropriate exclusions, especially to meet thresholds for determining suitability for point of care or other use-cases. In addition, greater use of blind test sets for validation would reduce bias from over-fitted machine learning models in MS based diagnostic testing. Furthermore, and especially for the less invasive sampling matrices, considerable work is required to harmonize and optimize methodologies so that features can be validated between labs.

## Funding

The authors would like to acknowledge 10.13039/501100000266EPSRC Fellowship Funding EP/R031118/1 and EP/P001440/1 in addition to funding by the 10.13039/501100003513University of Surrey and 10.13039/501100000268BBSRC BB/T00212/1.

## CRediT authorship contribution statement

**Matt Spick:** Conceptualization, Methodology, Software, Formal analysis, Writing – original draft, Visualization. **Holly M. Lewis:** Data curation, Validation. **Michael J. Wilde:** Data curation, Validation. **Christopher Hopley:** Conceptualization, Writing – review & editing. **Jim Huggett:** Conceptualization, Writing – review & editing. **Melanie J. Bailey:** Supervision, Project administration, Funding acquisition.

## Declaration of competing interest

The authors declare no competing interests.

## Appendix A Supplementary data

Supplementary material Image 1
