# Supplementary material for: Systematic review with meta-analysis of diagnostic test accuracy for COVID-19 by mass spectrometry
Source: Metabolism. 2022 Jan;126:154922. doi: 10.1016/j.metabol.2021.154922 (PMC8548837; doi:10.1016/j.metabol.2021.154922)
Supplement: Supplementary file 1 — Supplementary material [file mmc1.docx]

**Supplementary Material**


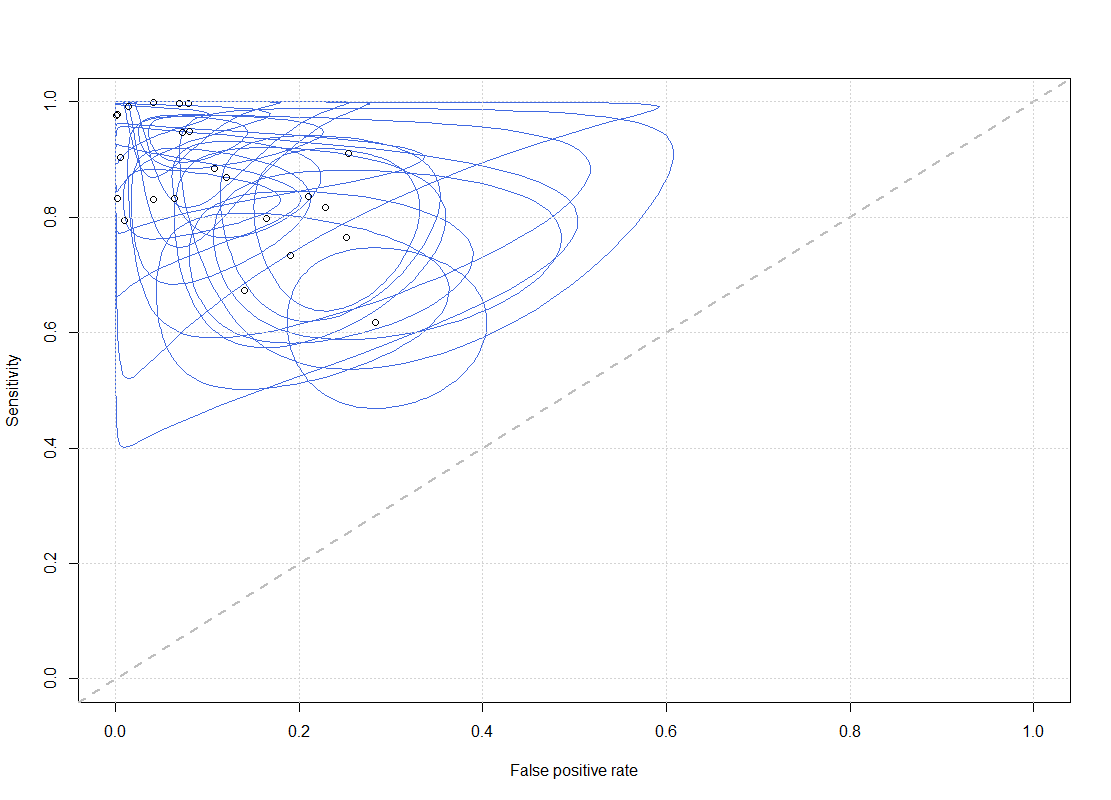


**Figure S1**: Sensitivity versus false positive rate (1 minus specificity) for reviewed studies plotted with 95% confidence intervals


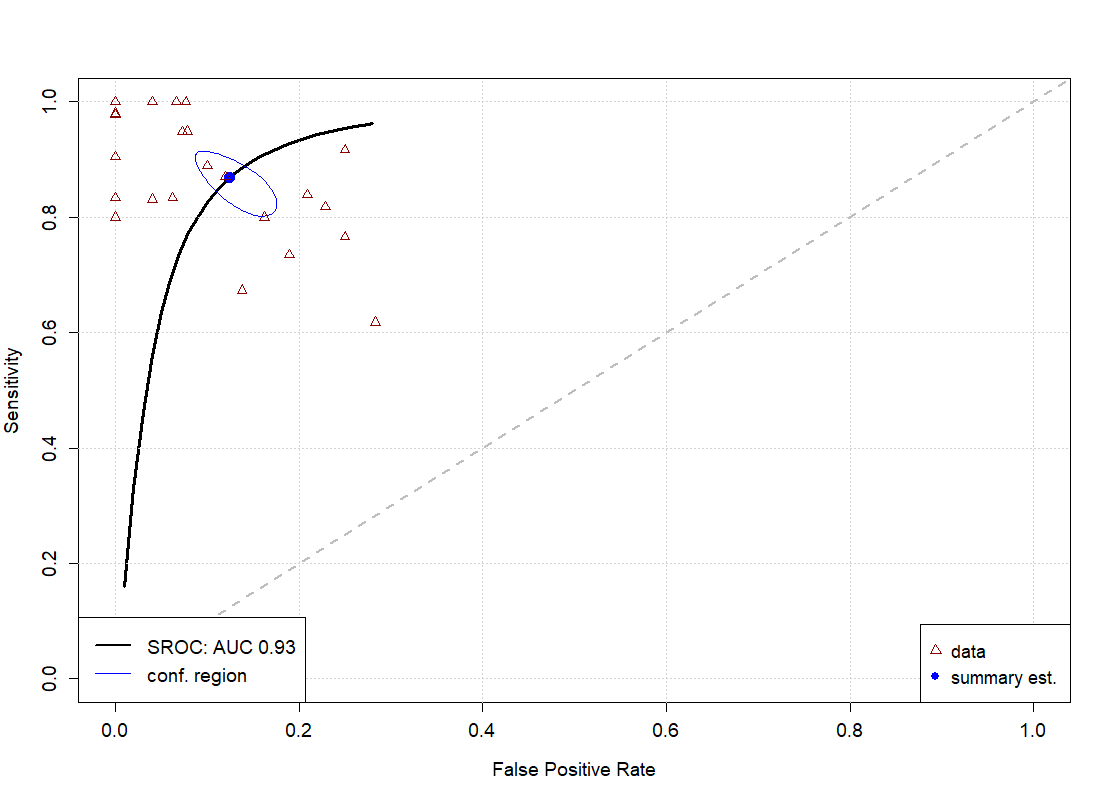


**Figure S2**: Summary Area Under Receiver Operating Curve for reviewed studies

**Table S1**: QUADAS-2 Questionnaire

| Domain | Questions |
| --- | --- |
| PATIENT SELECTION | BIAS - Was a consecutive or random sample of patients enrolled? |
|  | BIAS - Did the study avoid inappropriate exclusions, or were all patients recruited to the study included? For example were asymptomatic patients excluded? |
|  | BIAS - Was a case control design avoided? For example were the controls healthy and therefore mismatched? |
|  | APPLICABILITY – Is the test applicable to a clinical setting, i.e. could it distinguish COVID-19 participants in a triage type setting? |
| INDEX TEST | BIAS – Was the index (new) test done “blind” versus the reference test? |
|  | BIAS – Were any thresholds used in the index (new) test pre-set, or were they set to maximise sensitivity and specificity? |
|  | APPLICABILITY – Does the conduct of the index test or its interpretation differ from the review question, or is the index test directly designed for the review question? |
| REFERENCE STANDARD | BIAS – Is the reference standard 100% sensitive? |
|  | BIAS - Was the reference test done “blind” versus the index (new) test? |
|  | APPLICABILITY – Are there any concerns that the target condition identified by the reference standard may differ from the review question? |
| FLOW AND TIMING | BIAS – Was there a too long interval between index test and reference standard? |
|  | BIAS – Did all patients receive identical reference tests? |
